# Control over π-π stacking of heteroheptacene-based nonfullerene acceptors for 16% efficiency polymer solar cells

**DOI:** 10.1093/nsr/nwaa189

**Published:** 2020-08-25

**Authors:** Yunlong Ma, Dongdong Cai, Shuo Wan, Pan Yin, Pengsong Wang, Wenyuan Lin, Qingdong Zheng

**Affiliations:** State Key Laboratory of Structural Chemistry, Fujian Institute of Research on the Structure of Matter, Chinese Academy of Sciences, Fuzhou 350002, China; State Key Laboratory of Structural Chemistry, Fujian Institute of Research on the Structure of Matter, Chinese Academy of Sciences, Fuzhou 350002, China; State Key Laboratory of Structural Chemistry, Fujian Institute of Research on the Structure of Matter, Chinese Academy of Sciences, Fuzhou 350002, China; University of Chinese Academy of Sciences, Beijing 100049, China; State Key Laboratory of Structural Chemistry, Fujian Institute of Research on the Structure of Matter, Chinese Academy of Sciences, Fuzhou 350002, China; University of Chinese Academy of Sciences, Beijing 100049, China; State Key Laboratory of Structural Chemistry, Fujian Institute of Research on the Structure of Matter, Chinese Academy of Sciences, Fuzhou 350002, China; University of Chinese Academy of Sciences, Beijing 100049, China; State Key Laboratory of Structural Chemistry, Fujian Institute of Research on the Structure of Matter, Chinese Academy of Sciences, Fuzhou 350002, China; College of Chemistry, Fuzhou University, Fuzhou 350116, China; State Key Laboratory of Structural Chemistry, Fujian Institute of Research on the Structure of Matter, Chinese Academy of Sciences, Fuzhou 350002, China

**Keywords:** polymer solar cell, power conversion efficiency, nonfullerene acceptor, ladder‐type heteroheptacene, π–π stacking, charge carrier mobility

## Abstract

Nonfullerene acceptors are being investigated for use in polymer solar cells (PSCs), with their advantages of extending the absorption range, reducing the energy loss and therefore enhancing the power conversion efficiency (PCE). However, to further boost the PCE, mobilities of these nonfullerene acceptors should be improved. For nonfullerene acceptors, the π–π stacking distance between cofacially stacked molecules significantly affects their mobility. Here, we demonstrate a strategy to increase the mobility of heteroheptacene-based nonfullerene acceptors by reducing their π–π stacking distances via control over the bulkiness of lateral side chains. Incorporation of 2-butyloctyl substituents into the nonfullerene acceptor (M36) leads to an increased mobility with a reduced π–π stacking distance of 3.45 Å. Consequently, M36 affords an enhanced PCE of 16%, which is the highest among all acceptor-donor-acceptor-type nonfullerene acceptors to date. This strategy of control over the bulkiness of side chains on nonfullerene acceptors should aid the development of more efficient PSCs.

## INTRODUCTION

Having salient features of intrinsic flexibility, light weight, transparency and low-cost solution processability, bulk-heterojunction (BHJ) polymer solar cells (PSCs) are emerging as promising power supplies in applications such as portable electronics and building-integrated photovoltaic systems [1–8]. Over the past two decades, tremendous efforts have been devoted to PSCs thereby resulting in significantly improved power conversion efficiencies (PCEs) of over 17% for both single-junction and tandem devices [6,9,10]. In particular, development of novel donor and acceptor materials that can provide complementary absorptions, matched energy levels, favorable morphology as well as balanced and efficient charge transports is a major research focus [3,4,11]. For a long period of time, acceptor materials have been dominated by the fullerene derivatives (such as PC_61_BM and PC_71_BM), which exhibited PCEs over 11% when blended with appropriate low bandgap donor polymers [8,12–14]. However, attempts at further efficiency improvement of these fullerene-based PSCs have been fruitless because of the intrinsic weaknesses of the fullerene derivatives, such as weak or little absorption in the visible to near IR region, limited tunability of optical bandgaps and energy levels, and large exciton binding energy. Therefore, there is increasing interest in nonfullerene acceptors with an acceptor-donor-acceptor (A-D-A) structure because they may have tunable optical bandgaps and energy levels in a wider range, and smaller exciton binding energy. One representative example is 3,9-bis(2-methylene-(3-(1,1-dicyano-methylene)-indanone))-5,5,11,11-tetrakis(4-hexyl-phenyl)-dithieno[2,3-d : 2^′^,3^′^-d^′^]-*s*-indaceno[1,2-b : 5,6-b^′^]dithiophene (ITIC), which was first reported by Zhan and coworkers in 2015 [15]. When ITIC was blended with wide bandgap polymer donors, the corresponding PSCs afforded exciting PCEs of 9–12% [16–18]. Since then, many ITIC-derived nonfullerene acceptors have been developed to fine-tune the energy levels and morphology thereby leading to further improved PCEs approaching ∼13–15% [19–38].

For ITIC-derived nonfullerene acceptors, soluble side chains are needed to bond to the bridging atoms (carbon atom in most cases) of the ladder-type fused-ring cores. These side chains not only increase the solubility and film-forming characteristics of the resulting acceptors, but also suppress excessive molecular aggregation in the BHJ blend films [15]. However, the *sp*^3^-hybridized bridging atoms mean that the lateral side chains always stick out from the main π-conjugated backbone planes, enlarging their intermolecular π–π stacking distances, and thus lowering their instinct electron mobilities and limiting PCEs of the corresponding solar cells to some extent [39]. To minimize the unfavorable steric hindrance induced by the out-of-plane side chains, a new molecular design strategy for nonfullerene acceptors was thus proposed by introducing a nitrogen atom as the bridging atom to replace the traditional carbon atom in the central ladder-type donor core [39]. In contrast to the tetrahedral *sp*^3^-hybridized carbon atom, the *sp*^2^-hybridized nitrogen has a planar configuration and side chains can be functionalized on the *sp*^2^-hybridized nitrogen within the π-conjugated backbone plane. This coplanar configuration will facilitate close π–π stacking between cofacially stacked molecules, thus enhancing the charge carrier transport. Moreover, the stronger electron-donating capability of nitrogen compared with carbon atom can further enhance the intramolecular charge transfer (ICT) and thus reduce the bandgap of the nonfullerene acceptor, which is beneficial for improving the short-circuit current density (*J*_SC_) and therefore the PCE of PSC. However, only a few nitrogen-bridged ladder-type nonfullerene acceptors have been reported to date [39–41]. One of the most successful examples is the nonfullerene acceptor SN6IC-4F, which was first reported by Huang and co-workers [39]. By pairing with a wide bandgap polymer donor of PBDB-T, the optimized PSCs based on SN6IC-4F showed a high PCE of 13.2% [39]. The removal of the *sp*^3^-hybridized atoms in the conjugated backbones of nonfullerene acceptors can also be applied to A-D-A-D-A-type nonfullerene acceptors. For example, the Zou group developed a new family of nitrogen-bridged nonfullerene acceptors (Y-series) using an electron-deficient unit as the central core, and the best-performance acceptors showed initial PCEs over 15% [42,43]. The discovery of these Y-series nonfullerene acceptors greatly promoted the advances of organic solar cells, and since then many research groups have used these Y-series nonfullerene acceptors (especially Y6) to successfully achieve PSCs with PCEs over 16% [10,44–48]. The results suggest a promising strategy of designing nonfullerene acceptors using ladder-type conjugated backbones without the traditional *sp*^3^-hybridized carbon atoms. However, for the A-D-A-type nonfullerene acceptors, it is still challenging to achieve PSCs with PCEs approaching 16%.

The π–π stacking distance between cofacially stacked nonfullerene molecules greatly influences their charge carrier mobility. Theoretical calculation suggests that a shorter π–π stacking distance will potentially increase the charge transfer integral that represents the electronic wavefunction overlap between the adjacent molecules [49]. Thus, reducing the π–π stacking distance of the nonfullerene acceptor should be an effective way to improve its charge carrier mobility and photovoltaic performance. Nonfullerene acceptors with short lateral substituents are usually highly crystalline, which could lead to large-sized phase separation with donor materials thereby adversely affecting their photovoltaic performance. Whereas, nonfullerene acceptors with long lateral substituents would be less crystalline, which could lead to an increased π–π stacking distance and therefore a decreased charge carrier transport. It is noted that alkyl chains could not provide any charge carrier transport channels in devices. Therefore, for the nonfullerene acceptors free of the *sp*^3^-hybridized carbon atoms, a good balance between crystallinity and solubility is needed to improve the charge carrier mobility as well as the final photovoltaic performance. At the same time, nonfullerene acceptors should have a face-on molecular orientation to achieve good charge carrier transport in the vertical direction. Although the crystalline behavior and molecular orientation of nonfullerene acceptors have often been modulated via side chain engineering towards PSCs with improved photovoltaic performance [27,30], systematic investigations on effects of lateral substituents on the crystallinity, π–π stacking, molecular orientation, carrier transport and photovoltaic performance of nonfullerene acceptors are still rare, and are of great importance for developing high-performance photovoltaic materials. With these considerations in mind, in this work, we used ladder-type benzo[1,2-b : 4,5-b^′^]bis(4-*H*-dithieno[3,2-b : 2^′^,3^′^-d]pyrrole) as the donor core for efficient A-D-A-type nonfullerene acceptors without any *sp*^3^-hybridized bridging carbon atoms in the conjugated backbone. To alleviate the strong aggregation tendency of the resulting nonfullerene acceptors, four bulky branched alkyl chains were introduced, in which two pairs of side chains are located at the adjacent position in the backbone to prevent serious aggregation. To finely balance the molecular stacking and BHJ morphology, we systematically modulated the branched alkyl chain lengths, i.e. 2-ethylhexyl (M2), 2-butyloctyl (M36) and 2-decyltetradecyl (M38), of the resulting acceptor molecules. It was observed that M36 with medium-sized 2-butyloctyl side chains exhibited optimal aggregation behavior with a preferential face-on molecular orientation, the smallest π–π stacking distance of 3.45 Å, and the highest electron mobility in the BHJ film. In contrast, M2 with 2-ethylhexyl side chains and M38 with 2-decyltetradecyl side chains showed increased π–π stacking distances of 3.51 Å and 4.08 Å, respectively. As a result, the best-performance PSC based on M36 achieved an outstanding PCE of 16.00%, much higher than those based on M2 (11.16%) and M38 (8.89%). The initial PCE of 16.00% is comparable to those of the Y-series nonfullerene acceptors, and, to the best of our knowledge, M36 is the first A-D-A-type nonfullerene acceptor with a PCE approaching 16.00%.

## RESULTS AND DISCUSSION

**Scheme 1.**
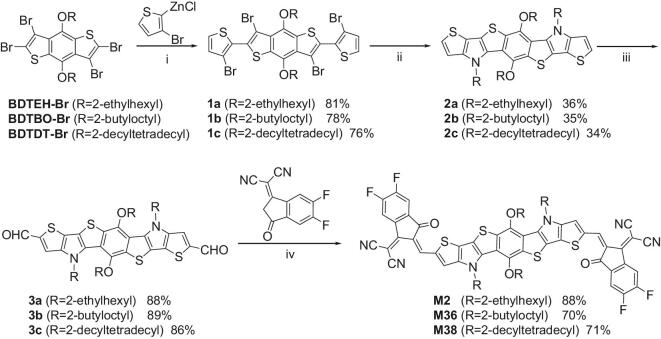
Synthesis of nonfullerene acceptors (M2, M36 and M38): (i) Pd(dppf)Cl_2_, reflux; (ii) 2-ethylhexylamine for 2a, 2-butyloctylamine for 2b, 2-decyltetradecylamine for 2c, Pd(dba)_2_, dppf, NaO*t*Bu, toluene, 110°C; (iii) ClCH_2_CH_2_Cl, 60°C; (iv) pyridine, CHCl_3_, 50°C.

The chemical structures and the synthetic route for the three nonfullerene acceptors (M2, M36 and M38) are shown in Scheme 1. The heptacyclic benzodi(dithienopyrrole) core was synthesized via Negishi coupling followed by Buchwald-Hartwig coupling, according to the procedure reported by Mitsudo *et al.* [50]. Then, the Vilsmeier-Haack reaction was used to functionalize these heptacyclic core units with two aldehyde groups. The final nonfullerene acceptors (M2, M36 and M38) were obtained through a Knoevenagel condensation reaction between 2-(5,6-difluoro-3-oxo-2,3-dihydro-1H-inden-1-ylidene)malononitrile (INCN2F) and corresponding aldehyde intermediates (3a–c). The detailed synthetic procedures and structural analyses, including ^1^H NMR, ^13^C NMR, high resolution mass spectrometry and element analysis were provided in the supplementary material. All the nonfullerene acceptors can be easily dissolved in common organic solvents such as chloroform and chlorobenzene.

**Figure 1.**
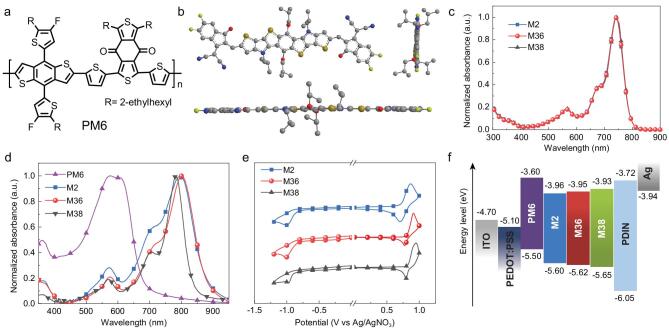
(a) Chemical structure of polymer donor PM6. (b) Optimized geometry of the nonfullerene acceptors calculated by the DFT calculation. Long branched alkyl chains are replaced by shorter isobutyl groups to simplify the calculations. (c) Normalized absorption spectra for the three acceptors in solutions. (d) Normalized absorption spectra for the three acceptors and polymer donor of PM6 in films. (e) Cyclic voltammograms of the three acceptors. (f) Energy level diagram of the materials used in this work.

The optimized geometries of the acceptors were calculated using the density functional theory (DFT) method at B3LYP/6–311G^**^ level. To simplify the DFT calculations, the long alkyl chains were replaced by short isobutyl groups. As shown in Fig. 1b, this type of nonfullerene acceptors have highly planar and straight π-conjugated backbones, which are beneficial to achieve a minimized intermolecular π–π stacking distance thereby improving the charge carrier mobility.

The ultraviolet-visible (UV-Vis) absorption spectra for M2, M36 and M38 in diluted solutions (5 × 10^−6^ M) as well as in thin films were measured (Fig. 1c and d), and the detailed optical parameters are summarized in Supplementary Table S1. In solution, the three nonfullerene acceptors exhibit almost identical absorption profiles with absorption peaks ranging from 740 to 743 nm (Fig. 1c), similar molar extinction coefficients and the same full width at half maximum (FWHM) value of 69 nm (Supplementary Table S1). These results can be explained by all three nonfullerene acceptors sharing the same π-conjugated backbone, and without noticeable aggregate formation in dilute solutions the lateral alkyl substituents can barely affect the electronic states of the nonfullerene acceptors. Compared with those in solution, the absorption spectra of the three acceptors in thin films display obviously bathochromically shifted absorption spectra (Fig. 1d), suggesting their formation of aggregate in the solid state. With increasing side chain length, the FWHM value of thin-film absorption decreases from 140 nm for M2, to 118 nm for M36, and finally to 84 nm for M38. The results suggest that the side chains could help to form more ordered molecular packing, which in turn reduces the broadening of absorption spectra in going from solution to thin film. At the same time, we note that, from solution to thin film, the absorption maxima of M2 and M36 exhibit considerable red-shifts by 52 and 57 nm, respectively, which are larger than the red-shift of 42 nm for M38 with longer side chains. The results suggest that side chains can dramatically affect the aggregate behavior of nonfullerene acceptors in solid state, which is important for photovoltaic application. The different shapes and peak wavelengths of thin-film absorption spectra for the three nonfullerene acceptors should be related to the different steric hindrances of the lateral side chains, which could significantly affect their π–π stacking

[36,51]. Similar results have also been reported in the Y-series nonfullerene acceptors [48]. The optical bandgaps (*E*_g_^opt^) for M2, M36 and M38 calculated from the absorption onsets of their thin films are 1.39, 1.39 and 1.47 eV, respectively. The absorption spectrum of the polymer donor PM6 (Fig. 1a) was also measured, and is shown in Fig. 1d. Not surprisingly, the BHJ active layers based on PM6 and the three acceptors provide a complementary absorption, covering a wide absorption range from 400 to 900 nm, which is beneficial to enhance *J*_SC_ values of the resulting PSCs.

The energy levels of the three acceptors in films were determined with use of cyclic voltammetry (CV) measurements with Ag/Ag^+^ as a reference. As shown in Fig. 1e and Supplementary Table S1, all the acceptors show similar highest occupied molecular orbital (HOMO) levels ranging from −5.60 to −5.65 eV and lowest unoccupied molecular orbital (LUMO) levels varying from −3.93 to −3.96 eV, suggesting a negligible effect of lateral alkyl chains on their electrochemical properties. Figure 1f shows the energy level diagram of the acceptors and polymer donor (PM6). It is noteworthy that the HOMO energy offsets between the acceptors and PM6 (0.10 eV for PM6:M2, 0.12 eV for PM6:M36, 0.15 eV for PM6:M38) are all less than 0.30 eV. These small offsets suggest the great potential of obtaining a small energy loss (*E*_loss_) and a large *V*_OC_ for the resulting PSCs, provided that the excitons generated from the acceptor component can be dissociated efficiently. Thus, we carried out photoluminescence (PL) measurements of the neat and blend films to investigate the exciton dissociation and photo-induced charge transfer of the donor: acceptor blends. As shown in Supplementary Fig. S1, PL intensities of the pure polymer donor or acceptors decrease dramatically upon incorporating the other component in the corresponding blend films, which suggests that the exciton dissociation and charge transfer between PM6 and the three acceptors are generally efficient.

**Figure 2.**
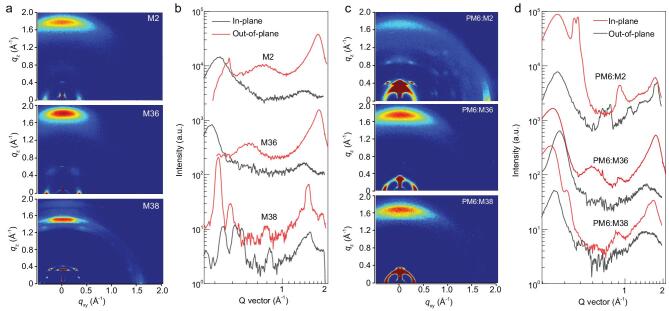
(a) 2D GIWAXS patterns and (b) the corresponding line-cuts of GIWAXS patterns for pure acceptor films. (c) 2D GIWAXS patterns and (d) the corresponding line-cuts of GIWAXS patterns for blend films.

To explore the molecular packing behaviors of the three acceptors (M2, M36 and M38) in neat films, grazing-incidence wide-angle X-ray scattering (GIWAXS) measurements were carried out (Fig. 2a and b, Supplementary Table S2). All three acceptors in neat films showed a preferential face-on orientation with respect to the substrate. In the out-of-plane direction, the (010) diffraction peaks of M2, M36 and M38 are located at 1.79, 1.82 and 1.54 Å^−1^, corresponding to π–π stacking distances of 3.51, 3.45 and 4.08 Å, respectively. The π–π stacking distance of 3.45 Å for M36 represents one of the smallest π–π stacking distances that has been achieved for nonfullerene acceptors [30,42,52]. To evaluate the crystallinity of nonfullerene acceptors in thin films, coherence lengths (*CLs*) for both the π–π stacking and lamellar stacking structures were estimated with use of Scherrer's equation, *CL *= 2π*K*/FWHM, where FWHM was calculated from the corresponding diffraction peak, and *K* = 0.9 [21]. As shown in Supplementary Table S2, increased *CL*s were obtained in both the π–π stacking and lamellar

stacking structures for the nonfullerene acceptors with the increasing lateral side chains. The more ordered molecular packing observed for the nonfullerene acceptor with longer side chains agrees with the corresponding thin-film absorption results we discussed previously. In addition, the significantly increased π–π stacking distance for M38 explains its abnormally blue-shifted absorption in thin film (in comparison with M36, and as shown in Fig. 1d). The GIWAXS results reconfirm the strong effect of lateral side chains on the π–π stacking of nonfullerene acceptors. Both too short and too long side chains are detrimental to the ordered and close π–π stacking, which can greatly influence the electron orbital overlap and therefore the charge transport.

**Figure 3.**
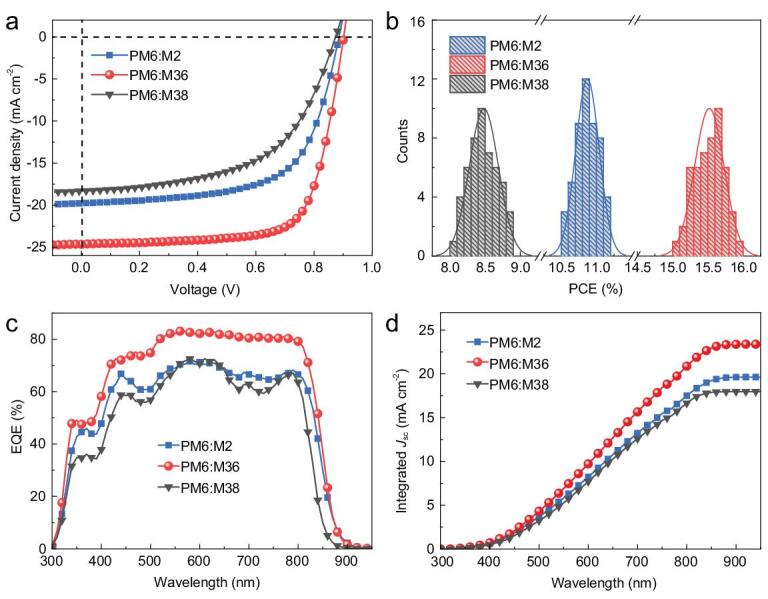
(a) *J−V* characteristics of the best-performance PSCs based on PM6:M2, PM6:M36 and PM6:M38. (b) PCE statistical distribution histograms of PSCs. (c) EQE spectra, and (d) the corresponding integrated curves for the best-performance PSCs.

To evaluate the photovoltaic performance of M2, M36 and M38, binary PSCs were fabricated with a conventional structure of indium tin oxide (ITO)/PEDOT:PSS/active layer/PDIN/Ag, where PEDOT:PSS is poly(3,4-ethylenedioxythiophene):poly(styrenesulfonate) and PDIN is a perylene diimide derivative functionalized with amino groups [53]. The benchmark polymer PM6 was selected as the donor polymer as a result of its complementary absorption (Fig. 1d) and matched energy levels with the three acceptors (Fig. 1f). The device performance was optimized by use of different fabrication conditions, including the donor:acceptor (D:A) weight ratio, solvent additive and thermal annealing. Detailed photovoltaic parameters are summarized in Supplementary Tables S3–5. The optimal active layers were achieved by spin-coating the PM6:acceptor (1 : 1, *w*/*w*) blend solution with a total concentration of 16 mg mL^−1^ in chloroform, and 0.5% (*v*/*v*) 1-chloronaphthalene was used as the solvent additive, followed by thermal annealing at 90°C for 5 min. The current density-voltage (*J* − *V*) curves of the best-performance PSCs based on PM6 and the three acceptors are shown in Fig. 3a, and the related device parameters are listed in Table 1.

**Table 1.** Photovoltaic properties of PSCs based on PM6:acceptors and SCLC charge carrier mobilities of the corresponding active layers.

Active layer	*V*_OC_ (V)	*J*_SC_ (mA cm^−2^)	FF (%)	PCE (%)^a^	*μ*_h_ (× 10^−4^ cm^2^ V^−1^ s^−1^)^b^	*μ*_e_ (× 10^−4^ cm^2^ V^−1^ s^−1^)^b^	*μ*_h_/*μ*_e_
PM6:M2	0.88	19.76	63.84	11.16 (10.87 ± 0.17)	6.62 (6.46 ± 0.17)	1.33 (1.21 ± 0.10)	4.98
PM6:M36	0.90	24.63	72.09	16.00 (15.52 ± 0.21)	10.70 (9.81 ± 0.53)	5.77 (5.60 ± 0.11)	1.85
PM6:M38	0.87	18.28	55.74	8.89 (8.48 ± 0.21)	4.20 (4.09 ± 0.09)	0.57 (0.55 ± 0.01)	7.38

^a^Average values with standard deviations were obtained from at least 50 independent devices. ^b^Average mobilities with standard deviations were obtained from eight independent devices.

Under the optimal device fabrication conditions, the best-performance device based on PM6:M2 yielded a PCE of 11.16%, with an open-circuit voltage (*V*_OC_) of 0.88 V, a *J*_SC_ of 19.76 mA cm^−2^ and a fill factor (FF) of 63.84%. Compared with the PM6:M2-based device, the PM6:M36-based PSC showed a much higher PCE of 16.00% with a *V*_OC_ of 0.90 V, a *J*_SC_ of 24.63 mA cm^−2^ and an FF of 72.09%. As for M38 bearing the longest side chains, the corresponding device afforded the lowest PCE of 8.89% among the three acceptors, which is mainly ascribed to its inferior *J*_SC_ (18.28 mA cm^−2^) and FF (55.74%). Figure 3b shows the histograms and corresponding Gaussian distribution of PCE counts for PM6:M2-, PM6:M36- and PM6:M38-based devices. To reconfirm the accuracy of the high PCE for the PM6:M36-based device, we sent an optimized device based on PM6:M36 to Fujian Metrology Institute (National PV Industry Measurement and Testing Center) in China for certification. They obtained an average PCE of 16.05% with a *V*_OC_ of 0.8931 V, a *J*_SC_ of 23.86 mA cm^−2^ and an FF of 75.35% (Supplementary material). To the best of our knowledge, the PCE of 16.05% is the highest for PSCs based on all A-D-A-type nonfullerene acceptors reported to date, and it is also comparable to those based on the Y-series (A-D-A-D-A-type) nonfullerene acceptors [42–44].

Figure 3c shows the external quantum efficiency (EQE) spectra of the three best-performance devices. We found that the photo response ranges of the M2- and M36-based devices are broader than that of the M38-based device, which is consistent with their absorption variation trends in thin films. Moreover, the M36-based device had the highest overall EQE values among the three best-performance PSCs. The *J*_SC_ values calculated from the EQE spectra (Fig. 3d) were determined to be 19.65, 23.41 and 18.00 mA cm^−2^ for the M2-, M36- and M38-based devices, respectively, which match well with the *J*_SC_ values obtained from the *J-V* measurements within 4.2% mismatches.

**Figure 4.**
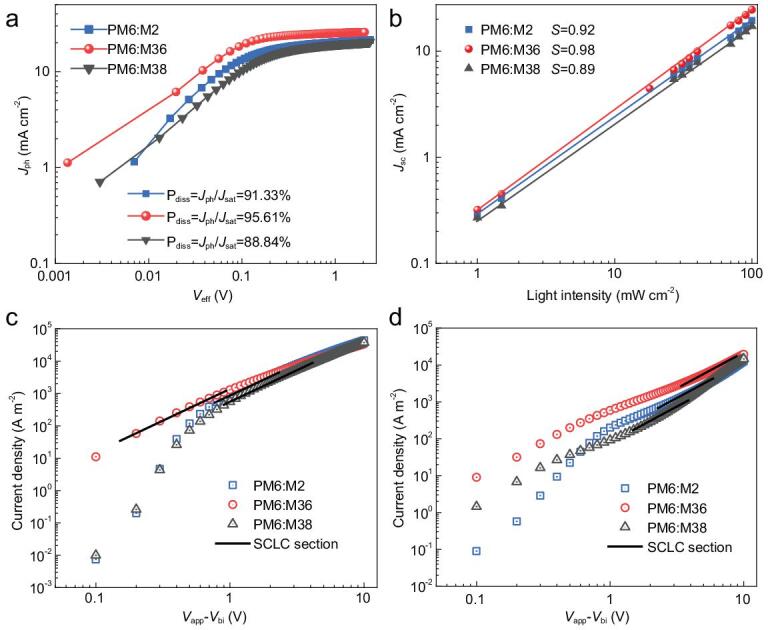
(a) Photocurrent density versus effective voltage (*J*_ph_ − *V*_eff_) characteristics for the best-performance devices under constant incident light intensity (AM1.5G,  100 mW cm^−2^). (b) Double logarithmic plots of *J*_SC_ as a function of incident light intensity for the best-performance devices. (c) *J-V* curves of hole-only and (d) electron-only devices based on PM6:M2, PM6:M36 and PM6:M38.

Devices based on the three acceptors produced similar high *V*_OC_s, ranging from 0.87 to 0.90 V, as expected from their similar LUMO levels. However, *J*_SC_ and FF values of the devices based on the three acceptors varied markedly. To elaborate on the factors leading to the large differences in the *J*_SC_ and FF values, we investigated the charge generation, transport and recombination behaviors of PSCs based on the three different acceptors. To probe the exciton dissociation process of the three best-performance PSCs, plots of photocurrent density (*J*_ph_) versus effective voltage (*V*_eff_) were measured and displayed in Fig. 4a. The exciton dissociation efficiency (*P*_diss_) can be determined from the ratio of *J*_ph_/*J*_sat_, where *J*_sat_ is saturation current density. Under short-circuit conditions, the ratios were 91.33%, 95.61% and 88.84% for the M2-, M36- and M38-based devices, respectively, indicating a much higher exciton dissociation efficiency for the M36-based device. This is consistent with the greatly enhanced PCEs for the M36-based PSCs. We then measured the devices by varying the light intensity (*P*_light_) from 1 to 100 mW cm^−2^ and analyzed the *P*_light_ dependence of *J*_SC_ to study the charge recombination in the three best-performance devices. As shown in Fig. 4b, by fitting the curve with the equation of *J*_SC_ ∝ *P*_light_*^S^*, the power-law exponent (*S*) values were 0.92, 0.98 and 0.89 for the M2-, M36- and M38-based devices, respectively. The largest *S* value for the M36-based device indicates the most suppressed charge recombination, partially contributing to its higher *J*_SC_ and FF values. To gain insight into the charge transport behaviors of PSCs, hole (*μ*_h_) and electron (*μ*_e_) mobilities of all the three BHJ blends were measured using the space charge limited current (SCLC) method. As depicted in Fig. 4c and d as well as in Table 1, PM6:M38 had hole- and electron-mobilities of 4.20 × 10^−4^ and 5.70 × 10^−5^ cm^2^ V^−1^ s^−1^, respectively, with a high *μ*_h_/*μ*_e_ ratio of 7.38. Such extremely unbalanced charge transport could lead to a severe charge recombination for the corresponding PSC device. By contrast, PM6:M2 not only afforded increased hole- and electron-mobilities of 6.62 × 10^−4^ and 1.33 × 10^−4^ cm^2^ V^−1^ s^−1^, respectively, but also had a more balanced *μ*_h_/*μ*_e_ ratio of 4.98, which can partly explain its improved *J*_SC_ and FF values in comparison with the M38-based counterparts. As for the PM6:M36-based devices, both the hole- and electron-mobilities were further improved simultaneously. The hole- and electron-mobilities of PM6:M36 increased to 1.07 × 10^−3^ and 5.77 × 10^−4^ cm^2^ V^−1^ s^−1^, respectively, with a most balanced *μ*_h_/*μ*_e_ ratio of 1.85, thus leading to the highest *J*_SC_ and FF as well as PCE values for the M36-based PSC among all the devices based on the three acceptors.

Morphology is essential to the performance of PSCs. To study the surface and bulk morphology of the blend films at the optimized conditions, atomic force microscopy (AFM) and transmission electron microscopy (TEM) measurements were performed. As illustrated in Supplementary Fig. S2, the root-mean-square roughness (*R*_q_) values are 11.90, 1.14 and 2.03 nm for the PM6:M2, PM6:M36 and PM6:M38 blend films, respectively. The smoothest surface for the M36-based blend implies the most homogeneous morphology, which would be favorable for forming good contact at the interface between the active layer and cathode, thereby facilitating the charge extraction. From the AFM phase images (Supplementary Fig. S2) and TEM images (Supplementary Fig. S3), the three blend films exhibit different aggregation features. The PM6:M2 blend film has many large-sized domains, which can be attributed to the relatively low solubility and a stronger aggregation tendency of M2. The excessive aggregates in active layers could serve as charge traps and thus increase the probability of the charge recombination. In the case of M38 with the longest branched alkyl chains, a less defined phase separation was found in its blend film, which is unfavorable for effective charge generation. In contrast, the PM6:M36 blend shows nanoscale phase-separated morphologies with fibrillar structures, which is favorable for efficient charge generation and transport. The morphologies of the three blend films are basically correlated with their photovoltaic properties (Table 1).

Two-dimensional GIWAXS was further used to investigate the molecular stacking and crystallization of the three BHJ active layers. For comparison purposes, the molecular packing behavior of neat PM6 film was also tested (Supplementary Table S2). PM6 shows a preferred face-on orientation, with a (010) π–π stacking peak at *q*_z_ = 1.72 Å^−1^ (*d *≈ 3.66 Å, where *d* is the π–π stacking distance) in the out-of-plane direction and a lamellar (100) diffraction peak at *q*_xy_ = 0.31 Å^−1^ (*d *≈ 20.21 Å) in the in-plane direction. The (010) and (100) *CL*s were calculated to be 1.75 and 5.68 nm, respectively. As shown in Fig. 2c and d, these blend films reveal distinct differences in the detailed microstructure and molecular orientation. For the PM6:M2 blend, quite a few scattering points are distributed over the 2D GIWAXS images, indicating the highly crystalline property. Moreover, the (010) diffraction peaks along the in-plane direction and out-of-plane direction are both obvious, suggesting coexistence of edge-on and face-on orientation textures for the PM6:M2 blend film. Unlike the PM6:M2 blend film, both the PM6:M36 and PM6:M38 blend films prefer face-on orientations, which would benefit efficient charge transport in the vertical direction across the active layers. Compared with the scattering data of single-component films, the PM6:M36 blend film exhibits combined diffraction features from both single-component materials, whereas the PM6:M38 blend film shows a PM6-dominated diffraction pattern. Such differences can be attributed to the discrepancy in the crystallization nature of M36 and M38 in the blend. The unbalanced crystallization properties of donor and acceptor in the PM6:M38 blend would result in an inefficient and unbalanced charge transport, which agrees with the data measured by the SCLC method. The change trend of π–π stacking distance for the three blends is similar to that for the corresponding pure acceptor films. The π–π stacking distance decreases first from *d *≈ 3.63 Å (*q*_z_ = 1.73 Å^−1^) for PM6:M2, to 3.58 Å (*q*_z_ = 1.75 Å^−1^) for PM6:M36, and then increases to 3.74 Å (*q*_z_ = 1.68 Å^−1^) for PM6:M38. However, the (010) *CL* in the out-of-plane direction gradually decreases from 3.01 nm, to 2.16 nm and to 2.09 nm for the PM6:M2, PM6:M36 and PM6:M38 blends, respectively, indicating that the extension of the alkyl chains would inevitably destroy the strong molecular π–π stacking in the blend film. The ordered crystalline structure together with the closest π–π stacking distance for M36 is beneficial for enhanced charge transport and restrained charge recombination, thus accounting for the best *J*_SC_, FF and PCE values for the PM6:M36-based device. The GIWAXS results generally agree with the charge transport properties of the blend films.

## CONCLUSION

In conclusion, we have developed a novel family of A-D-A-type nonfullerene acceptors (M2, M36 and M38) based on a ladder-type heteroheptacene aromatic core flanked with varied alkyl chains. The effect of the lateral branched alkyl chains on the optical, π–π-stacking, charge carrier transport and photovoltaic properties of the nonfullerene acceptors were systematically investigated. Use of a short lateral side chain of 2-ethylhexyl, resulted in the nonfullerene acceptor (M2) exhibiting a short π–π stacking distance of 3.51 Å with a strong tendency of aggregation. Use of a long side chain of 2-decyltetradecyl, resulted in the nonfullerene acceptor (M38) showing a long π–π stacking distance of 4.08 Å. While the use of the medium-sized 2-butyloctyl side chain led to the shortest π–π stacking distance of 3.45 Å for the resulting nonfullerene acceptor (M36), which represents one of the smallest π–π stacking distances achieved for nonfullerene acceptors. In addition to the difference in the π–π stacking distance, both the long (2-decyltetradecyl) and the short (2-ethylhexyl) side chains resulted in heteroheptacene-based nonfullerene acceptors with blue-shifted absorption in comparison with that for 2-butyloctyl-based acceptor (M36). As a result, when blended with a benchmark copolymer of PM6, M36 afforded an enhanced PCE of 16.00% with an increased *J*_SC_ of 24.63 mA cm^−2^ and an improved *μ*_e_ of 5.77 × 10^−4^ cm^2^ V^−1^ s^−1^ in comparison with M2 (PCE = 11.16%; *J*_SC _= 19.76 mA cm^−2^; *μ*_e_ = 1.33 × 10^−4^ cm^2^ V^−1^ s^−1^) and M38 (PCE = 8.89%; *J*_SC _= 18.28 mA cm^−2^; *μ*_e_ = 5.7 × 10^−5^ cm^2^ V^−1^ s^−1^). Furthermore, the 16.00% PCE is the highest among all A-D-A-type nonfullerene acceptors, to the best of our knowledge. We believe that by tuning the bandgap, choosing other donor materials and optimizing the fabrication condition, the performance of PSCs based on these heteroheptacene-based A-D-A-type nonfullerene acceptors could be increased further. The ladder-type heteroheptacene-based backbone without any *sp*^3^-hybridized bridging carbon atoms in combination with lateral side chains of optimal bulkiness affords an efficient molecular design strategy for nonfullerene acceptors with reduced π–π stacking distances, thereby leading to PSCs with improved charge carrier mobilities, increased *J*_SC_ values and therefore enhanced PCEs.

## Supplementary Material

nwaa189_Supplemental_File
