# Supplementary material for: Control over π-π stacking of heteroheptacene-based nonfullerene acceptors for 16% efficiency polymer solar cells
Source: Natl Sci Rev. 2020 Aug 25;7(12):1886–95. doi: 10.1093/nsr/nwaa189 (PMC8288506; doi:10.1093/nsr/nwaa189)
Supplement: nwaa189_Supplemental_File [file nwaa189_supplemental_file.docx]

**Supplementary material**

**Control over π-π stacking of heteroheptacene-based nonfullerene acceptors for 16% efficiency polymer solar cells**

Yunlong Ma^1^, Dongdong Cai^1^, Shuo Wan^1,2^, Pan Yin^1,2^, Pengsong Wang^1,2^, Wenyuan Lin^1,3^ and Qingdong Zheng^1,*^

^1^ State Key Laboratory of Structural Chemistry, Fujian Institute of Research on the Structure of Matter, Chinese Academy of Sciences, Fujian 350002, China
^2^ University of Chinese Academy of Sciences, Beijing 100049, China

^3^ College of Chemistry, Fuzhou University, Fuzhou, Fujian 350116, China

^∗^ Corresponding author. E-mail: qingdongzheng@fjirsm.ac.cn

**Materials and Instruments**

PM6 and 2-(5,6-difluoro-3-oxo-2,3-dihydro-1H-inden-1-ylidene)malononitrile (INCN2F) were purchased from Solarmer Materials Inc. and Suna Tech Inc., respectively. Other solvents and reagents were purchased from Aldrich Inc., Adamas-beta Ltd., Suna Tech Inc. and Energy Chemical, and used directly without further purification unless otherwise specified. ^1^H and ^13^C NMR spectra were recorded at 400 MHz on a Bruker AVANCE-400 spectrometer using tetramethylsilane (TMS) as the internal standard. High-resolution mass spectroscopy measurements were determined on a Thermo Scientific QExactive mass spectrometer. Elemental analyses were carried out using a Vario EL-Cube elemental analyzer. UV-vis absorption spectra were obtained from a Lambda 365 UV-vis spectrophotometer. Photoluminescence spectra were taken on an Edinburgh Instrument FLS 920. All the film samples for absorption and photoluminescence measurements were annealed at 90 ^o^C for 5 min. The surface topography of the active layer was recorded by atomic force microscopy (AFM) operated in the tapping mode. Transmission electron microscopy (TEM) images were recorded on a F20 Tecnai instrument operating at 200 kV of accelerating voltage. Cyclic voltammetry (CV) measurement was performed on a CHI 604E electrochemical workstation with a three-electrode cell in a nitrogen bubbled 0.1 M tetrabutylammonium hexafluorophosphate (Bu_4_NPF_6_) solution in acetonitrile at a scan rate of 100 mV s^-1^ at room temperature. Platinum wire, Ag/AgNO_3_ (0.1 M AgNO_3_ in acetonitrile) and platinum plate were used as the counter electrode, reference electrode and working electrode, respectively. The Ag/AgNO_3_ reference electrode was calibrated using a ferrocene/ferrocenium redox couple as an external standard, whose oxidation potential is set at -4.80 eV with respect to zero vacuum level. Under this condition, the onset oxidation potential of ferrocene was -0.02 V *versus* Ag/Ag^+^. The small molecule films were coated on the Pt plate electrode by dipping the electrode into corresponding solutions and then drying. The HOMO energy level was calculated using the equation of *E*_HOMO_ = -(*φ*_ox_ + 4.82) (eV). The LUMO level was calculated using the equation of *E*_LUMO_ = -(*φ*_red_ + 4.82) (eV).

**Synthesis and Characterization**

Compounds BDTEH-Br, BDTBO-Br, BDTDT-Br, **1a-c** and **2a-c** were prepared according to previously reported methods [S1-S2].

***Synthesis of compound 1a***: To a solution of 2,3-dibromothiophene (2.42 g, 10 mmol) in Et_2_O (15 mL) was added dropwise *n*-butyllithium (2.5 M, 4 mL) at -78 °C. After being stirred at this temperature for 1 h, a solution of zinc chloride (1 M in THF, 10 mL) was dropped slowly with a syringe. The resulting mixture was warmed to 0 °C and stirred for another 1 h. Then, BDTEH-Br (1.89 g, 2.50 mmol) and Pd(dppf)Cl_2_ (0.18 g, 0.25 mmol) were added to the mixture. The mixture was stirred at reflux for 24 h. After being cooled to room temperature, aq. NH_4_Cl solution was added to the resulting mixture which was then extracted with diethyl ether. The combined organic layers were dried, filtered, concentrated *via* rotary evaporation, and purified by silica gel chromatography with petroleum ether:CH_2_Cl_2_ (10:1) as an eluent, yielding compound **1a** as a yellow solid (1.86 g, 81%). ^1^H NMR (400 MHz, CDCl_3_, δ): 7.56 (d, *J* = 5.2 Hz, 2H), 7.19 (d, *J* = 5.2 Hz, 2H), 4.16 (d, *J* = 5.6 Hz, 4H), 2.09 (m, 2H), 1.70-1.35 (m, 16H), 1.09 (t, *J* = 7.2 Hz, 6H), 0.92 (t, *J* = 7.2 Hz, 6H).

***Synthesis of compound 1b***: Compound **1b** was synthesized according to the same procedure as that for **1a**. The pure product of compound **1b** was obtained as a yellow oil (2.32 g, 78%). ^1^H NMR (400 MHz, CDCl_3_, ppm): 7.53 (d, *J* = 5.2 Hz, 2H), 7.16 (d, *J* = 5.2 Hz, 2H), 4.14 (d, *J* = 6.8 Hz, 4H), 2.06 (m, 2H), 1.69-0.90 (m, 44H).

***Synthesis of compound 1c***: Compound **1c** was synthesized according to the same procedure as that of **1a**. The pure product of compound **1c** was obtained as a yellow oil (2.16 g, 76%). ^1^H NMR (400 MHz, CDCl_3_, ppm): 7.50 (d, *J* = 5.2 Hz, 2H), 7.15 (d, *J* = 5.2 Hz, 2H), 4.10 (d, *J* = 6.8 Hz, 4H), 2.03 (m, 2H), 1.67-1.35 (m, 92H).

***Synthesis of compound 2a***: To a suspension of sodium *tert*-butoxide (3.84 g, 40.00 mmol) in 20 mL of anhydrous toluene were added compound **1a** (1.52 g, 2.00 mmol), Pd(dba)_2_ (0.11 g, 0.20 mmol) and dppf (0.44 g, 0.80 mmol). The resulting mixture was deoxygenated with nitrogen for 30 min, and then, 2-ethylhexylamine (0.78 g, 6.00 mmol) was added. The mixture was stirred at reflux for 12 h under nitrogen. After cooling to room temperature, the mixture was poured into deionized water and extracted with dichloromethane. The combined organic phase was washed with water and dried with MgSO_4_. After evaporation of solvent, the residue was purified by column chromatography with petroleum ethers as eluent yielding compound **2a** as a light-yellow oil (0.51 g, 36%). ^1^H NMR (400 MHz, CDCl_3_, δ): 7.30 (d, *J* = 5.2 Hz, 2H), 7.20 (d, *J* = 5.2 Hz, 2H), 4.83 (d, *J* = 6.8 Hz, 4H), 4.10 (d, *J* = 6.8 Hz, 4H), 2.17-2.03 (m, 4H), 1.80-0.73 (m, 56H). HRMS (MALDI) m/z: calcd. for C_50_H_72_N_2_O_2_S_4_, 860.4477; found, 860.4473.

***Synthesis of compound 2b****:* Compound **2b** was synthesized according to the same procedure as that of **2a**. The pure product of compound **2b** was obtained as a yellow oil (0.42 g, 35%). ^1^H NMR (400 MHz, CDCl_3_, δ): 7.20 (d, *J* = 5.2 Hz, 2H), 7.10 (d, *J* = 5.2 Hz, 2H), 4.80 (d, *J* = 6.8 Hz, 4H), 4.02 (d, *J* = 6.8 Hz, 4H), 2.11-1.92 (m, 4H), 1.80-0.73 (m, 88H). HRMS (MALDI) m/z: calcd. for C_66_H_104_N_2_O_2_S_4_, 1084.6981; found, 1084.6973.

***Synthesis of compound 2c****:* Compound **2c** was synthesized according to the same procedure as that of **2a**. The pure product of compound **2c** was obtained as a yellow oil (0.38 g, 34%). ^1^H NMR (400 MHz, CDCl_3_, δ): 7.19 (d, *J* = 5.2 Hz, 2H), 7.07 (d, *J* = 5.2 Hz, 2H), 4.72 (d, *J* = 6.8 Hz, 4H), 3.99 (d, *J* = 6.8 Hz, 4H), 2.12-1.99 (m, 4H), 1.66-0.69 (m, 184H). HRMS (MALDI) m/z: calcd. for C_114_H_200_N_2_O_2_S_4_, 1757.4493; found, 1757.4482.

***Synthesis of compound 3a***: In a dry two neck round-bottomed flask, compound **2a** (0.50 g, 0.58 mmol) was dissolved in 20 mL of 1,2-dichloroethane and placed under nitrogen atmosphere. The solution was cooled to 0 °C and stirred while phosphorus oxychloride (1.81 g, 11.60 mmol) and DMF (0.84 g, 11.60 mmol) were added successively. The mixture was stirred for 1 h at 0 °C, and then stirred for 12 h at 60 °C. After the reaction, the mixture was cooled to room temperature and poured into ice water, neutralized with Na_2_CO_3_, and then extracted with dichloromethane. The combined organic layer was washed with water and brine, dried over anhydrous MgSO_4_. After the removal of solvent, the residue was purified by column chromatography on silica gel using petroleum ether/dichloromethane (1:1) as the eluent, yielding compound **3a** as an orange crystalline solid (0.47 g, 88%). ^1^H NMR (400 MHz, CDCl_3_, δ): 9.98 (s, 2H), 7.76 (s, 2H), 4.82 (d, *J* = 6.8 Hz, 4H), 4.03 (d, *J* = 6.8 Hz, 4H), 2.10-1.96 (m, 4H), 1.77-0.73 (m, 58H). ^13^C NMR (100 MHz, CDCl_3_, δ): 183.2, 145.8, 144.1, 141.6, 135.6, 123.7, 120.7, 119.8, 115.6, 78.7, 52.5, 40.6, 40.5, 30.3, 29.9, 29.2, 28.1, 23.7, 23.5, 23.2, 23.0, 14.2, 13.9, 11.3, 10.6; HRMS (MALDI) m/z: calcd. for C_52_H_72_N_2_O_4_S_4_, 916.4369; found, 916.4374.

***Synthesis of compound 3b***: Compound **3b** was synthesized according to the same procedure as that for compound **3a**. A yellow crystalline solid of compound **3b** was isolated in 89% yield. ^1^H NMR (400 MHz, CDCl_3_, δ): 9.97 (s, 2H), 7.75 (s, 2H), 4.78 (d, *J* = 6.8 Hz, 4H), 4.02 (d, *J* = 6.8 Hz, 4H), 2.14-2.00 (m, 4H), 1.67-0.71 (m, 88H). ^13^C NMR (100 MHz, CDCl_3_, δ): 183.2, 145.9, 144.1, 141.7, 141.6, 135.6, 123.8, 120.7, 119.8, 115.7, 79.0, 52.7, 39.2, 39.1, 32.0, 31.7, 31.2, 30.9, 30.8, 30.6, 29.9, 29.5, 29.3, 28.2, 27.0, 25.9, 23.2, 22.9, 22.9, 22.6, 14.2, 14.1, 13.9; HRMS (MALDI) m/z: calcd. for C_68_H_104_N_2_O_4_S_4_, 1140.6873; found, 1140.6870.

***Synthesis of compound 3c***: Compound **3c** was synthesized according to the same procedure as that for compound **3a**. The pure product of compound **3c** was obtained as a yellow oil (0.41 g, 86%). ^1^H NMR (400 MHz, CDCl_3_, δ): 9.93 (s, 2H), 7.70 (s, 2H), 4.76 (d, *J* = 6.8 Hz, 4H), 3.98 (d, *J* = 6.8 Hz, 4H), 2.10-1.97 (m, 4H), 1.63-0.82 (m, 184H). ^13^C NMR (100 MHz, CDCl_3_, δ): 183.1, 145.8, 144.0, 141.7, 141.6, 135.6, 123.8, 120.5, 119.8, 115.6, 79.0, 52.6, 39.2, 39.1, 32.0, 31.3, 30.8, 30.2, 30.0, 29.9, 29.8, 29.7, 29.6, 29.5, 29.4, 27.1, 27.0, 26.0, 22.8, 14.2; HRMS (MALDI) m/z: calcd. for C_116_H_200_N_2_O_4_S_4_, 1813.4385; found, 1813.4382.

***Synthesis of M2***: Compound **3a** (0.10 g, 0.11 mmol) and 2-(5,6-difluoro-3-oxo-2,3-dihydro-1H-inden-1-ylidene)malononitrile (0.20 g, 0.88 mmol) were dissolved in 20 mL of anhydrous chloroform. The mixture was deoxygenated with nitrogen for 30 min before 0.1 mL of pyridine was added. The resulting mixture was stirred at 50 °C for 8 h under nitrogen. After cooling to room temperature, the reaction mixture was poured into methanol and the precipitate was filtered off. The crude product was then purified by silica gel column using petroleum ether/dichloromethane (1:1 by volume) as the eluent. The pure product of **M2** was obtained as a dark blue crystalline solid (0.12 g, 88%). ^1^H NMR (400 MHz, CDCl_3_, δ): 8.98 (s, 2H), 8.56 (m, 2H), 7.93 (s, 2H), 7.72 (t, *J* = 7.2 Hz, 2H), 4.80 (d, *J* = 7.6 Hz, 4H), 4.06 (m, 4H), 2.13-2.07 (m, 4H), 1.75-0.99 (m, 44H), 0.81 (m, 6H), 0.74 (t, *J* = 7.2 Hz, 6H); ^13^C NMR (100 MHz, CDCl_3_, δ): 185.9, 158.7, 155.0, 152.9, 148.0, 145.2, 144.8, 138.9, 137.2, 136.9, 136.6, 134.5, 132.6, 126.9, 120.7, 120.1, 117.1, 114.9, 114.7, 112.6, 79.1, 68.4, 52.7, 40.6, 40.3, 30.1, 29.7, 29.1, 27.9, 23.5, 23.3, 23.1, 22.9, 14.1, 13.8, 11.2, 10.5; HRMS (MALDI) m/z: calcd. for C_76_H_76_N_6_O_4_F_4_S_4_, 1340.4742; found, 1340.4750. Anal. calcd for C_76_H_76_N_6_O_4_F_4_S_4_: C 68.03, H 5.71, N 6.26; found: C 68.07, H 5.66, N 6.27.

***Synthesis of M36***: Compound **M36** was synthesized according to the same procedure as that for **M2**. A dark blue crystalline solid of **M36** was isolated in 70% yield. ^1^H NMR (400 MHz, CDCl_3_, δ): 8.98 (s, 2H), 8.54 (m, 2H), 7.92 (s, 2H), 7.71 (t, *J* = 7.6 Hz, 2H), 4.77 (d, *J* = 7.6 Hz, 4H), 4.05 (d, *J* = 6.8 Hz, 4H), 2.15-2.01 (m, 4H), 1.69-0.71 (m, 88H). ^13^C NMR (100 MHz, CDCl_3_, δ): 185.8, 158.6, 155.6, 153.0, 148.0, 145.3, 144.9, 138.9, 137.2, 136.9, 136.5, 134.6, 132.6, 127.0, 120.6, 120.1, 117.2, 114.9, 114.6, 112.6, 79.5, 68.3, 52.9, 39.3, 38.9, 31.9, 31.6, 31.2, 30.9, 30.6, 30.4, 29.8, 29.5, 29.2, 27.9, 27.0, 25.8, 23.2, 22.9, 22.7, 22.6, 14.2, 14.0, 13.8. HRMS (MALDI) m/z: calcd. for C_92_H_108_N_6_O_4_F_4_S_4_, 1564.7246; found, 1564.7235. Anal. calcd for C_92_H_108_N_6_O_4_F_4_S_4_: C 70.56, H 6.95, N 5.37; found: C 70.70, H 6.92, N 5.33.

***Synthesis of M38***: Compound **M38** was synthesized according to the same procedure as that for **M2**. A dark blue crystalline solid of **M38** was isolated in 71% yield. ^1^H NMR (400 MHz, CDCl_3_, δ): 8.99 (s, 2H), 8.57 (m, 2H), 7.94 (s, 2H), 7.70 (t, *J* = 7.6 Hz, 2H), 4.78 (d, *J* = 7.6 Hz, 4H), 4.05 (d, *J* = 6.8 Hz, 4H), 2.13-2.02 (m, 4H), 1.67-0.83 (m, 184H). ^13^C NMR (100 MHz, CDCl_3_, δ): 185.8, 158.7, 155.9, 153.1, 147.9, 145.3, 144.9, 138.9, 137.2, 136.9, 136.6, 134.5, 132.7, 127.1, 120.9, 120.1, 117.1, 114.9, 114.6, 112.4, 79.6, 68.4, 52.9, 39.3, 38.9, 34.0, 31.9, 31.2, 30.6, 30.2, 30.0, 29.8, 29.7, 29.6, 29.4, 27.1, 25.8, 22.7, 23.1, 22.9, 22.5, 14.1, 14.0, 13.3. HRMS (MALDI) m/z: calcd. for C_140_H_204_N_6_O_4_F_4_S_4_, 2237.4758; found, 2237.4778. Anal. calcd for C_140_H_204_N_6_O_4_F_4_S_4_: C 75.09, H 9.18, N 3.75; found: C 75.19, H 9.12, N 3.76.

**PSC device fabrication and characterization**

The PSC devices were fabricated with the configuration of ITO/PEDOT:PSS/ active layer/PDIN/Ag. ITO-coated glass substrates were cleaned by ultrasonicating sequentially in detergent, deionized water, acetone, and isopropanol for 10 min each and then dried in an oven at 80 ^o^C for 12 h. Then, the ITO glass substrates were subjected to ultraviolet/ozone treatment at room temperature for 15 min. The filtered PEDOT:PSS solution (Baytron PVP AI 4083 from H. C. Starck) was spin-coated onto the cleaned ITO substrates at 3500 rpm, followed by baking at 140 ºC for 15 min in air. Subsequently, the substrates were transferred into a N_2_-filled glove box for spin-coating the active layer. The active layer (ca. 150 nm) was then spin-coated from the blend solutions (total concentration of 16 mg mL^-1^) with 0.5 % (vol. %) 1-chloronaphthalene (CN) as additive, which were prepared at 50 ^o^C in chloroform. The mixed solutions were spin-coated on the top of the PEDOT:PSS layer at 3000 rpm for 45 s, followed by thermal annealing at 90 °C for 5 min. PDIN methanol solution (2.0 mg/mL) was then spin-coated on the active layer at 3000 rpm for 30 s to afford a buffer layer with a thickness of ca. 10 nm. Finally, 100 nm of Ag top electrode was deposited onto the PDIN buffer layer through shadow masks by thermal evaporation at a pressure of 1.0 × 10^-4^ Pa. The deposition rate and film thickness were monitored with a quartz crystal sensor. The active area of the devices was 4 mm^2^. After an encapsulation by epoxy kits (general purpose, Sigma-Aldrich) in the glovebox, the devices were illuminated through their ITO sides.

The current density-voltage (*J*-*V*) characteristics were measured using a Keithley 2400 Source-Measure Unit. An Oriel Sol3A simulator (Newport) was used as a light source. The light intensity was calibrated to 100 mW cm^-2^ by a NREL certified silicon reference cell. More than 8 devices were measured to provide the average PCEs of PSCs. EQE data were taken by using the QE/IPCE measurement kit (QE-PV-SI) from Newport.

**Hole- and electron-only device fabrication and characterization**

Hole- and electron- mobilities were measured using the space charge limited current (SCLC) method. Hole-only devices were fabricated with a architecture of ITO/PEDOT:PSS/active layer/MoO_3_/Ag, while electron-only devices were constructed with a architecture of ITO/ZnO/active layer/Ca/Al. The active layers were prepared using the same method as that used for the best-performance PSC fabrication. Device areas were fixed at 4 mm^2^. The current density (*J*) was measured by a Keithley 2440 source measurement unit. The SCLC hole/electron mobilities were calculated according to the following equation:

$J=\frac{9\varepsilon_{0}\varepsilon_{r}\mu V^{2}}{8L^{3}}$ (1)

Where *J* is the current density (A m^-2^), *ε*_0_ is the free-space permittivity (8.85 × 10^-12^ F m^-1^), *ε*_r_ is the relative dielectric constant of the active layer material usually 2-4 for organic semiconductors, herein we used a relative dielectric constant of 3, *μ* is the mobility of hole or electron, *V* is the voltage drop across the SCLC device (*V* = *V*_app_-*V*_bi_, where *V*_app_ is the applied voltage to the device and *V*_bi_ is the built-in voltage due to the difference in the work function of two electrodes, in the hole- and electron-only devices, the *V*_bi_ values are 0.5 and 0.7 V, respectively), and *L* is the thickness of the active layer. The film thickness was determined by a Bruker Dektak XT surface profilometer. The hole- or electron-mobilities were calculated from the slopes of the *J*^1/2^-*V* curves.

**GIWAXS characterization**

GIWAXS measurements were performed at 13A beamline of National Synchrotron Radiation Research Center (NSRRC, Hsinchu). Ceshigo Research Service (www.ceshigo.com) provided the technical support for the GIWAXS measurements. Samples were prepared on PEDOT:PSS-coated Si substrates using the identical blend solutions as those for the best-performance PSCs. All samples for GIWAXS measurements were radiated at 12.13 keV X-ray with an incident angle of 0.10-0.15°. The coherence length was estimated by the Scherrer equation: CL = 2πK/FWHM, where FWHM is the full width at half-maximum of the peak and K is a shape factor (0.9 was used here).


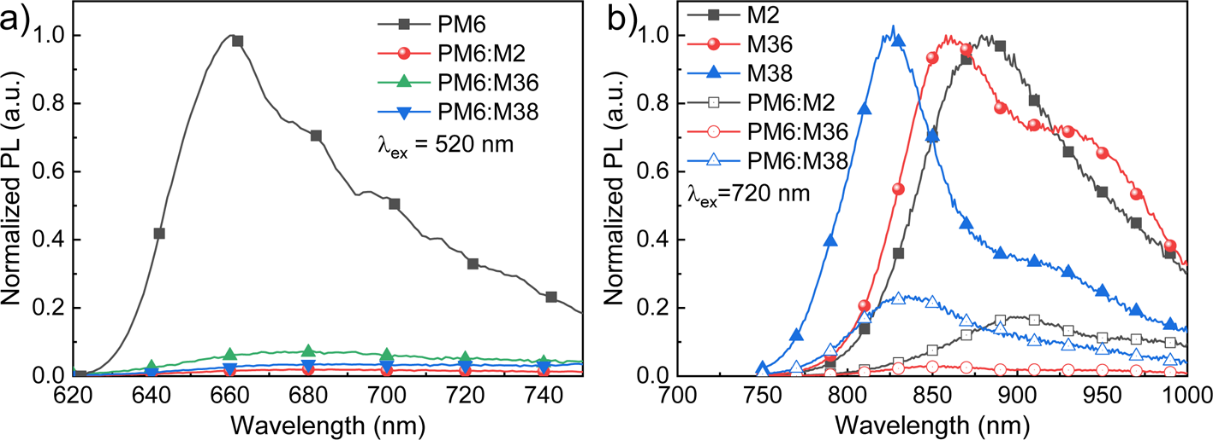


**Figure S1**. Normalized photoluminescence spectra of M2, M36, M38 and PM6 neat films and PM6:M2, PM6:M36 and PM6:M38 blend films.

**Figure S2**. AFM height images of PM6:M2 (a), PM6:M36 (b) and PM6:M38 (c) blend films; AFM phase images of PM6:M2 (d), PM6:M36 (e) and PM6:M38 (f) blend films.


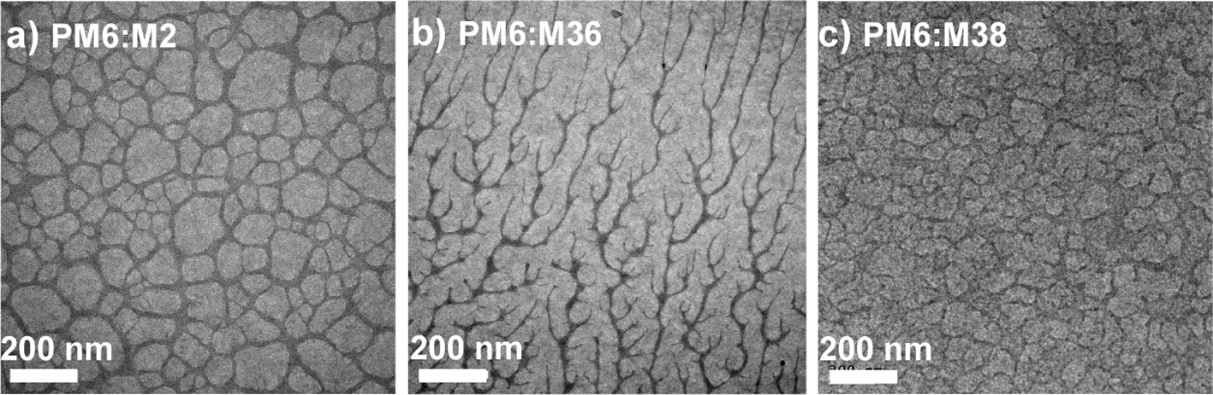


**Figure S3**. TEM images of PM6:M2 (a), PM6:M36 (b) and PM6:M38 (c) blend films.

**Table S1**. Optical and electrochemical properties of M2, M36 and M38.

| Molecules | ε  [10^5^ M^-1^ cm^-1^] | λ_max_^solution^ [nm]^a^ | λ_max_^film^  [nm]^a^ | *E_g_^opt^* [eV]^b^ | HOMO [eV]^c^ | LUMO [eV]^c^ |
| --- | --- | --- | --- | --- | --- | --- |
| M2 | 2.33 | 742 (69) | 794 (140) | 1.39 | -5.60 | -3.96 |
| M36 | 2.38 | 743 (69) | 800 (118) | 1.39 | -5.62 | -3.95 |
| M38 | 2.45 | 740 (69) | 782 (84) | 1.47 | -5.65 | -3.93 |

^a^ In the parentheses are full width at half maximum values; ^b^ Optical bandgap determined by *λ*_onset_ in thin films; ^c^ Measured by cyclic voltammetry.

**Table S2**. Summarized parameters of the ordered structures.

| Samples^a^ | π-π stacking | | Lamellar stacking | |
| --- | --- | --- | --- | --- |
|  | *d*-spacing (Å) | CL (Å) (FWHM)^b^ | *d*-spacing (Å) | CL (Å) (FWHM)^b^ |
| M2 | 3.51 | 22.54 (0.251 Å^-1^) | 17.28 | 58.94 (0.096 Å^-1^) |
| M36 | 3.45 | 23.78 (0.238 Å^-1^) | 19.72 | 95.88 (0.059 Å^-1^) |
| M38 | 4.08 | 42.50 (0.133 Å^-1^) | 16.56 | 169.17 (0.033 Å^-1^) |
| PM6:M2 | 3.63 | 30.10 (0.188 Å^-1^) | 21.58 | 67.19 (0.084 Å^-1^) |
| PM6:M36 | 3.58 | 21.59 (0.262 Å^-1^) | 20.52 | 93.21 (0.061 Å^-1^) |
| PM6:M38 | 3.74 | 20.91 (0.270 Å^-1^) | 22.84 | 106.58 (0.053 Å^-1^) |
| PM6 | 3.66 | 17.46 (0.324 Å^-1^) | 20.21 | 56.77 (0.997 Å^-1^) |
| ^a^ (010) diﬀraction peak along the *q*_z_ axis, (100) diﬀraction peak along the *q*_xy_ axis; ^b^ Coherent length (CL) estimated from the Scherrer equation (CL = 2π*K*/FWHM, *K* = 0.9). | | | | |

**Table S3**. Photovoltaic properties of PM6:M36-based PSCs with different blend ratios.^a^

| PM6:M36 (by weight) | *V*_oc_ [V] | *J*_sc_ [mA/cm^2^] | FF [%] | PCE [%] |
| --- | --- | --- | --- | --- |
| 1:0.8 | 0.89 | 23.72 | 70.66 | 14.93 (14.76 ± 0.14)^b^ |
| 1:1 | 0.90 | 24.63 | 72.09 | 16.00 (15.52 ± 0.21)^c^ |
| 1:1.2 | 0.90 | 24.27 | 70.71 | 15.40 (15.06 ± 0.25)^b^ |

^a^ The PM6:M36 blends were dissolved in chloroform with 0.5 vol % CN, and the active layers were annealed at 90 ^o^C for 5 min; ^b^ The average PCEs with standard deviations in the parentheses are based on 8 devices; ^c^ The average PCE with a standard deviation in the parentheses is based on 50 devices.

**Table S4**. Photovoltaic properties of PM6:M36-based PSCs with different amounts of CN additive.^a^

| CN (vol %) | *V*_oc_ [V] | *J*_sc_ [mA/cm^2^] | FF [%] | PCE [%] |
| --- | --- | --- | --- | --- |
| 0 | 0.89 | 23.52 | 66.29 | 13.84 (13.68 ± 0.24)^b^ |
| 0.3 | 0.89 | 23.83 | 69.79 | 14.84 (14.77 ± 0.06)^b^ |
| 0.5 | 0.90 | 24.63 | 72.09 | 16.00 (15.52 ± 0.21)^c^ |
| 1.0 | 0.90 | 24.29 | 71.64 | 15.68 (15.12 ± 0.21)^b^ |

^a^ The PM6:M36 blends (1:1 by weight) were dissolved in chloroform, and the active layers were annealed at 90 ^o^C for 5 min; ^b^ The average PCEs with standard deviations in parentheses are based on 8 devices; ^c^ The average PCE with a standard deviation in the parentheses is based on 50 devices.

**Table S5**. Photovoltaic properties of PM6:M36-based PSCs with different annealing temperatures.^a^

| Temperature [^o^C] | *V*_oc_ [V] | *J*_sc_ [mA/cm^2^] | FF [%] | PCE [%] |
| --- | --- | --- | --- | --- |
| 25 | 0.91 | 22.56 | 66.83 | 13.59 (13.29 ± 0.23)^b^ |
| 80 | 0.90 | 24.18 | 71.18 | 15.43 (15.19 ± 0.22)^b^ |
| 90 | 0.90 | 24.63 | 72.09 | 16.00 (15.52 ± 0.21)^c^ |
| 100 | 0.90 | 23.97 | 71.34 | 15.39 (15.28 ± 0.11)^b^ |

^a^ PM6:M36 blends (1:1 by weight) were dissolved in chloroform with 0.5 vol % CN, and the annealing time was set at 5 min; ^b^ The average PCEs with standard deviations in parentheses are based on 8 devices; ^c^ The average PCE with a standard deviation in the parentheses is based on 50 devices.


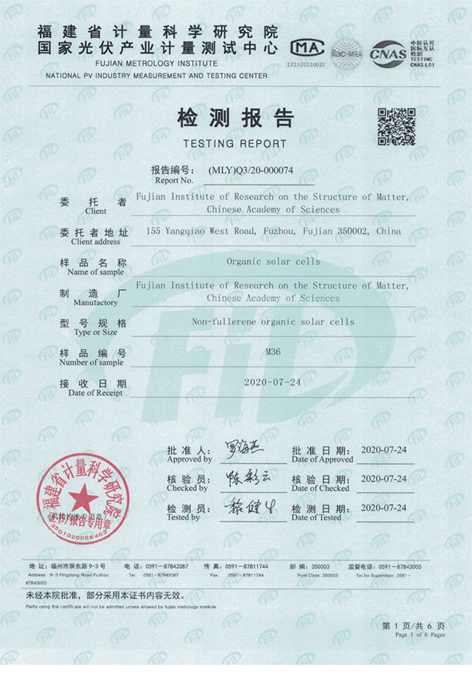

**References**

Rf

1. Son HJ, Wang W and Xu T, *et al*. Synthesis of fluorinated polythienothiophene-co-benzodithiophenes and effect of fluorination on the photovoltaic properties. *J Am Chem Soc*, 2011; **133**: 1885-94.
2. Mitsudo K, Shimohara S and Mizoguchi J, *et al*. Synthesis of nitrogen-bridged terthiophenes by tandem buchwald-hartwig coupling and their properties. *Org Lett*, 2012; **14**: 2702-5.
